# Predicting and mapping malaria under climate change scenarios: the potential redistribution of malaria vectors in Africa

**DOI:** 10.1186/1475-2875-9-111

**Published:** 2010-04-23

**Authors:** Henri EZ Tonnang, Richard YM Kangalawe, Pius Z Yanda

**Affiliations:** 1 Institute of Resource Assessment, University of Dar Es Salaam, PO Box 35097, Dar es Salaam, Tanzania

## Abstract

**Background:**

Malaria is rampant in Africa and causes untold mortality and morbidity. Vector-borne diseases are climate sensitive and this has raised considerable concern over the implications of climate change on future disease risk. The problem of malaria vectors (*Anopheles *mosquitoes) shifting from their traditional locations to invade new zones is an important concern. The vision of this study was to exploit the sets of information previously generated by entomologists, e.g. on geographical range of vectors and malaria distribution, to build models that will enable prediction and mapping the potential redistribution of *Anopheles *mosquitoes in Africa.

**Methods:**

The development of the modelling tool was carried out through calibration of CLIMEX parameters. The model helped estimate the potential geographical distribution and seasonal abundance of the species in relation to climatic factors. These included temperature, rainfall and relative humidity, which characterized the living environment for *Anopheles *mosquitoes. The same parameters were used in determining the ecoclimatic index (EI). The EI values were exported to a GIS package for special analysis and proper mapping of the potential future distribution of *Anopheles gambiae *and *Anophles arabiensis *within the African continent under three climate change scenarios.

**Results:**

These results have shown that shifts in these species boundaries southward and eastward of Africa may occur rather than jumps into quite different climatic environments. In the absence of adequate control, these predictions are crucial in understanding the possible future geographical range of the vectors and the disease, which could facilitate planning for various adaptation options.

**Conclusion:**

Thus, the outputs from this study will be helpful at various levels of decision making, for example, in setting up of an early warning and sustainable strategies for climate change and climate change adaptation for malaria vectors control programmes in Africa.

## Background

The Anopheles mosquitoes are the worldwide known vectors of malaria. In Africa, three *Anopheles *species: *Anopheles gambiae, Anopheles arabiensis *and *Anopheles funestus *are considered to be the major vectors most responsible for malaria transmission. The first two species belong to a group called *A. gambiae *complex, which is considered to be the most efficient malaria vector in the world [1]. Actual and future geographical distribution of these mosquito species is vital for malaria control strategic planning.

Pioneer work on malaria vector distribution [2,3] only reflected entomologists' opinion rather than the ecological niche of *Anopheles*. Lindsay *et al *[4] attempted the prediction for *Anopheles *species occurrence in Africa using climatic and distributional data. Coetzee *et al *[5] mapped African malaria vector species based on actual collections. The genetic algorithm for rule-set prediction (GARP) model was used to predict the geographical and ecological distribution of three species of the *A. gambiae *complex (*A. gambiae, A. arabiensis*, and *Anopheles quadriannulatus*). The approach was based on relating ecological niches of species point occurrence data to electronic maps of relevant ecological dimensions in order to produce rules that describe the potential distribution of the species [1].

Early hypothesis about climate change and vector-borne diseases in general proposed that increased temperature and precipitation could facilitate the emergence and persistence of *Anopheles *mosquitoes [6]. Furthermore, vector-borne diseases, such as malaria, would respond most promptly to localized warming events. Expected mosquitoes ranges increase with increased temperature, towards temperate regions, has been suggested [7]. A recent study by Lafferty [8] has added into the long-standing debate on how global warming will affect the prevalence and distribution of human vector-borne diseases. This author's analysis suggested that *Anopheles *distribution range shifts are more likely to occur than range expansions. It is then clear that one of the major threats for the current functioning of the world is the uncertainty about the effects of global climate change. Working knowledge of where malaria vectors specially occur and will potentially occur in the future under climate change scenarios is very essential for the malaria control programme managers and policy makers. Entomologists and malariologists throughout the African continent have collected distributional and biological data. This study aims to make use of these records to increase the understanding and produce potential future distributional maps for two members of the *A. gambiae *complex; *A. gambiae *and *A. arabiensis *under different climate change scenarios. The CLIMEX model, which is a platform, designed to infer species responses to climate from observations on their geographical distribution and seasonal phenology was used for the effect [9,10].

## Methods

### Data sets

The distributional records for malaria vectors used in this study came from the ARMA/MARA website [11], recent scientific publications and reports [12-14]. The meteorological data included: the average minimum daily temperature, the average maximum daily temperature, the average monthly rainfall, and the average daily relative humidity at 9 am and at 3 pm. This database was made with selected locations worldwide as well as interpolated climatic grids (5° × 5°) provided by the global change community through the Climatic Research Unit (CRU) in Norwich UK. The stations coverage is denser over the more populated parts of the world. The observation began with small number of stations during the 1850s, but increases to over 3,000 stations during the 1951-90 period.

### Assumptions

The African climate is diverse and varied. It is diverse because it ranges from humid equatorial regimes, through seasonally arid tropical regimes, to sub-tropical Mediterranean type. By this diversity, it demonstrates different degrees of spatial variability, particularly with regard to rainfall [15]. The temperatures vary greatly throughout the continent and each country has unique climatic conditions [15]. The altitude sometimes also moderates the climate. Based on these considerations, the African continent was divided into subsequent climatic zones:

- The band of latitudes centred on the equator was considered as the equatorial zone and the winter and summer seasons were defined with reference to the equatorial zone and the day of the year.

- To the north of the equatorial zone, summer was defined as the days from March 2 - September 30, with winter being the rest of the year. Reinforcing the same argument, south of the equatorial zone, the situation was the reverse.

### Climate change scenarios

Two major reasons guided the choice for the climate change scenarios; the first was linked to African climate as mentioned earlier and the second, the response of *Anopheles *mosquitoes to temperature and rainfall. The temperature, rainfall and humidity are important determinant factors for the *Anopheles *distribution. A little increase in temperature leads to an increase in the mosquito's development and the adult frequency of blood feeding [16]. The *Anopheles *breeding and survival are determined and characterized by rainfall and humidity. In relation to these criteria the following scenarios were chosen:

a) Scenario 1: A rise of 2°C Africa wide temperature, and 10% increase of summer rainfall and 10% decrease in winter rainfall.

b) Scenario 2: A 0.1°C rise in summer and winter maximum and minimum temperatures per degree of latitude, and a 10% increase in rainfall in summer, and 10% decrease in winter.

c) Scenario 3: A rise of 4°C Africa wide temperature, and 20% increase of summer rainfall and 20% decrease in winter rainfall.

### Model

Many theories accounting for the abundance, distribution, change and variation of species population have been proposed. They can broadly be divided in two classes; the first class regrouped those that view climate either directly or indirectly through its interactions with species-specific characters, as the major determining factor [17], and the second class gathered those that view multi-species interactions such as competition and predation as being the paramount [18] component for species distribution. The conceptual framework of the model used in this study was based on the first school of thought that stipulated that climate most likely has a major influence on the species abundance.

The platform applied is called CLIMEX. The model theory is generally based on the species estimated responses to temperature and moisture. The CLIMEX model estimates are founded on the assumption that if you know where a species lives, you can infer what climatic conditions it can tolerate. In other words, CLIMEX attempts to mimic the mechanisms that limit species geographical distributions and determine their seasonal phenology and to a lesser extent their relative abundance [9,10]. It derives weekly and annual indices that describe the responses of a nominated species to temperature and moisture. Furthermore, this model assumes that a species at a given location experience two seasons during a year, one with population growth and the other with population decline. These are referred to as growth or survival and stress seasons, respectively. The overall climatic suitability of a nominated location for a given organism is provided by an Ecoclimatic Index (EI), which combines the annual potential for population growth (GI_A_), with the annual stresses that limit survival during the unfavourable season (SI) and with the limiting interacting factor between stresses (SX) [19]. These indices are calculated as follows:(1)

Where: TI_W _and MI_w _are the weekly temperature and moisture respectively, 52 is the number of weeks in a year. CS, DS, HS and WS are the annual cold, dry, heat and wet stress indices respectively. CDX, CWX, HDX and HWX are the annual cold-dry, cold-wet, hot-dry and hot-wet stress interactions indices respectively.

The locations with positive values for EI are suitable for a species, and the larger the value of EI, the more suitable the location. As few climates are 100% suitable throughout the year, the value of EI will rarely reach its potential, thereby limiting the maximum value of EI [19].

### Model building, prediction test and special analysis

In building the model, the usually observed geographical distribution data from African countries belonging to the sub-Saharan region was mapped in accordance with each chosen *Anopheles *species population growth rate, the developmental threshold temperature and moisture levels. The CLIMEX model parameters were repeatedly adjusted and the function "*Compare location*", which describes the potential geographical distribution of a species, as limited by climate was re-run until the estimated potential *Anopheles *species range best matched the observed or known distribution.

In order to test the model's predictive capability, it was initially intended to use independent data; meaning records that do not have any connection with the information applied, for model parameters estimation. However, this intention encountered difficulties as the two species (*A. gambiae *and *A. arabiensis*) under prediction only occur in Africa and in no other location in the world. Soper and Wilson [20] recorded the establishment of one species of the *A. gambiae *complex in north-eastern part of Brazil around 1930 to 1940 to have been via accidental introduction. Therefore, the opportunity of using South America for the developed model validation became possible and practical. The parameters estimated from the building exercise were then used to run the "*Compare location*" function in South American region for predicting the *Anopheles *species potential distribution in north-eastern parts of Brazil. This and the building exercises were successively repeated by slightly adjusting parameters until a 'good fit' for the known distribution in Africa and mentioned location in South America, was obtained respectively.

The treatment of climate change was thereafter performed for the African continent. Best-matched parameters were fitted for mapping the *Anopheles *species under current conditions before the climate change scenarios were successively stated. The "compare location" was once again run and the EI for the *A. gambiae *and *A. arabiensis *were evaluated under current and the chosen climate change scenarios respectively. The differences between the values of EI for individual climate change scenario and the current scenario was performed for each *Anopheles *species. The obtained results were then transferred to Arc-GIS 9.2 package in order to facilitate the organization, manipulation, visualization, and for geospatial analysis.

## Results

### Potential current and future distribution of malaria vectors in Africa

**Figure 1:**
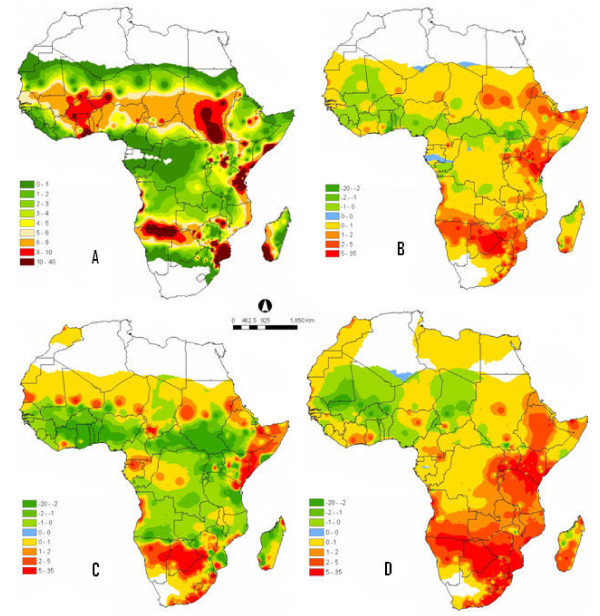
**(A) Distribution of *A. arabiensis *under current climate, the map was constructed using the ecoclimatic indices (EI) obtained from CLIMEX parameters in Table 1**. (B) Distribution of *A. arabiensis *illustrating species ranges shifts under climate change scenario 1. The map was developed from the difference between the values EI for the predicted future *A. arabiensis *distribution obtained when applying scenario 1 criteria and the distribution under current climate (A) in Africa. (C) Distribution of *A. arabiensis *illustrating species ranges shifts under climate change scenario 2. The map was developed from the difference between the values EI for the predicted future *A. arabiensis *distribution obtained when applying scenario 2 criteria and the distribution under current climate (A) in Africa. (D) Distribution of *A. arabiensis *illustrating species ranges shifts under climate change scenario 3. The map was developed from the difference between the values EI for the predicted future *A. arabiensis *distribution obtained when applying scenario 3 criteria and the distribution under current climate (A) in Africa

**Figure 2:**
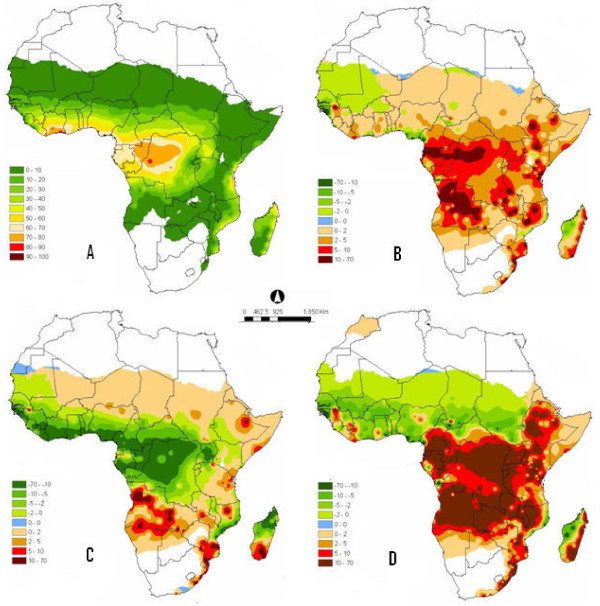
**(A) Distribution of *A. gambiae *under current climate, the map was constructed using the ecoclimatic indices (EI) obtained from CLIMEX parameters in Table 1**. (B) Distribution of *A. gambiae *illustrating species ranges shifts under climate change scenario 1. The map was developed from the difference between the values EI for the predicted future *A. gambiae *distribution obtained when applying scenario 1 criteria and the distribution under current climate (A) in Africa. (C) Distribution of *A. gambiae *illustrating species ranges shifts under climate change scenario 2. The map was developed from the difference between the values EI for the predicted future *A. gambiae *distribution obtained when applying scenario 2 criteria and the distribution under current climate (A) in Africa. (D) Distribution of *A. gambiae s *illustrating species ranges shifts under climate change scenario 3. The map was developed from the difference between the values EI for the predicted future *A. gambiae *distribution obtained when applying scenario 3 criteria and the distribution under current climate (A) in Africa.

The CLIMEX parameters (Table 1) were inferred from field and laboratory, or were estimated iteratively through manual adjustment until the model predictions matched the observed records well. The values of the EI show that the climate is most favourable for *A. arabiensis *within the drier savannah areas of the continent (Figure 1a). In the opposite, the most suitable region for *A. gambiae *is the wet tropical region (Figure 2a). Both figures represent the simulations using parameter values presented in Table 1. These illustrations are generally in agreement with previous conclusions [4,5], which described *A. arabiensis *and *A. gambiae *to be favourable in warmer and wetter environments or habitats respectively. Poor model fit in few localities may be due to the quality and insufficiencies of the African climate data used. Several countries were represented by only one or two stations and thus it can be said that the data used for such areas are coarse and may contain uncertainties that vary over small spatial scale.

**Table 1:** CLIMEX parameters values for African malaria vectors belonging to the *Anopheles gambiae *complex

	Values	
Parameter designation	*A. gambiae*	*A. arabiensis*
**Moisture parameters (proportion of soil moisture holding capacity)**		
Lower threshold of soil moisture (SM0)	0.35	0.15
Lower limit of optimal range of soil moisture (SM1)	0.70	0.40
Upper limit of optimal range of soil moisture (SM2)	1.50	0.60
Upper threshold of soil moisture (SM3)	2.50	0.80
		
**Temperature parameters (°C)**		
Lower threshold of temperature for population growth (DVO)	15	18
Lower optimal temperature for population growth (DV1)	28	30
Upper optimal temperature for population growth (DV2)	35	38
Upper threshold temperature for population growth (DV3)	40	44
		
**Dry stress indices**		
Soil moisture dry stress (proportion of soil holding capacity) (SMDS)	0.260	0.300
Rate of accumulation of dry stress (HDS)	-0.006	-0.001
		
**Wet stress indices**		
Soil moisture wet stress (proportion of soil holding capacity) (SMWS)	2.50	0.900
Rate of accumulation of wet stress (HWS)	0.20	0.003
		
**Heat stress indices**		
Threshold of heat stress (TTHS)	40.000	44.0000
Rate of accumulation of heat stress (TTHS)	0.001	0.0002
		
**Cold stress indices**		
Temperature threshold of cold stress (TTCS)	2.000	2.000
Rate of accumulation of cold stress (THCS)	-1.000	-1.000
Degree-days threshold of cold stress (DTCS)	25.000	15.000
Rate of accumulation of cold stress linked to degree-days (DHCS)	-0.002	-0.001
		
**Cold-wet stress indices**		
Degree-days threshold of cold-wet stress (DTCW)	30.000	-
Moisture threshold of cold-wet stress (MTCW)	0.100	-
Rate of accumulation of cold-wet stress (PCW)	0.001	-

The obtained maps (Figures 1b-c) and (Figures 2b-c) are for the difference between the values of EI of each climate change scenario and the current climate for *A. arabiensis *and *A. gambiae *respectively. Figure 1b describes the values of EI for climate change Scenario 1 minus the values of EI for *A. arabiensis *under current climate. Usually, the EI values are ranged between 0 and 100. An EI value close to 0 indicates that the location is not suitable for long-term survival of the species where as an EI value of 100 is equated to an ideal condition. However, some of the legends have shown negative EI values, this is because 'smaller' and positive EI values were subtracted from 'bigger' values. The goal of this exercise was to identify regions with either reduction or addition/surplus in suitability for the vectors, and evaluate their percentage.

All chosen climate change scenarios demonstrated that "under business as usual condition" the western and some region of central part of Africa might, with time, loose their habitat suitability for both studied *Anopheles *species. Under climate change scenario 1, 2 and 3 the West African region will become less suitable for both species ranges, whereas the malaria vectors may be shifting towards the eastern and southern regions of the continent. In contrast, the southern and eastern part of the continent might become more favourable for the development of the African malaria vectors.

In the legends, the rectangles (0 - 0) represent unchanged zones in terms of species suitability. In all circumstances, these regions are few, proving that changes in African malaria vectors distribution under rising temperature and rainfall variability is real. The EI values vary from (0 - 40) and from (0 - 100) and (-70 - 70) for *A. arabiensis *and *A. gambiae *respectively. This discrepancy is due to the changes on the dynamics of climate characteristics in savannah areas compared to wet regions. The savannah is characterized by a distinct dry season, which is generally in the winter and the rainy season in the summer months. During the dry seasons some rivers recede into pools and streams dry up. These pools may serve as breeding sites for malaria vectors.

**Figure 3:**
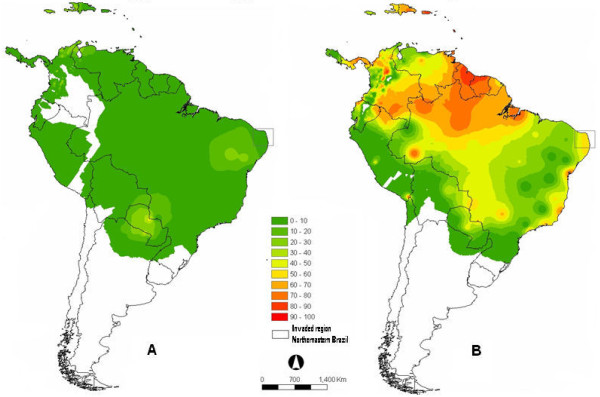
**(A) Projected suitable areas of *A. arabiensis *in South America**. (B) Projected suitable areas of *A. gambiae *in South America. The square in north eastern of Brazil indicates the area where a member of *A. gambiae *complex was established and later eradicated around 1930.

It is convenient that predictive models for continental species distribution should be tested using independent data sets from the same species ranges in another continent different from the one used for model development. This is because a species requires almost identical factors everywhere on the planet Earth in order to survive. The methodology mentioned a retrospective event of the 1930s, where a member of the *A. gambiae *complex was imported to Brazil. During that period, the *A. gambiae *was yet to be recognized as a complex of multiple species, and the real identity of the member of the complex that was responsible for malaria transmission in the area remained unidentified, and speculations have been made that the species that invaded the area was either *A. gambiae *or *A. arabiensis*. In validating the model, it was suggested and established that the *A. gambiae *species was likely responsible for the deadly malaria outbreak in southern eastern Brazil around the 1930s (Figure 3).

## Discussion

The modelling approach enabled results to be directly exported to Arc-GIS 9.2 for standardised spatial analysis. The representation of the results became sensible showing species ranges rather than simple point estimates which usually incorporates much of the uncertainty surrounding the input parameters, and allow unclear conclusions to be drawn. Another advantage of the applied methodology is that it does allow the user to obtain useful predictions with minimum information. This was demonstrated through the fact that the database on actual distribution of malaria vectors in Africa is filled with blank areas where no species identification has been published. Nevertheless, satisfactory outputs were obtained.

The results of this study can be compared with other analyses: Lindsay *et al *[4], Coetzee *et al *[5] and Levine *et al *[1]. The first authors used climatic and distributional data to predict the African malaria vectors occurrence, but this resulted in maps with limitations. The *A. arabiensis *was projected to occur in South Africa, within an arid region with no rivers. The discrepancy in such results is obvious, as the mosquitoes cannot survive in areas without possibility for breeding sites. The second study by Coetzee *et al *[5] only mapped climatic factors comprising the actual distribution of malaria vectors. No attempt was made in predicting future distribution of the species. The third authors [1] used ecological niche modelling to predict the potential distribution of *A. gambiae *complex. However, their results projected that *A. arabiensis *will be redistributed across most of central Africa, which is generally humid. This is unlikely to be realistic because the *A. arabiensis *mostly inhabits semiarid environments.

These results generally agree with current observed maps of malaria vectors. When applying different climate change scenario analyses, they displayed different possible changes in suitability within the continent. These analyses have proven that changes in ranges for both *Anopheles *species under climate change are likely to be incremental and nonlinear. The present results have shown that shifts in these species boundaries southwards and eastwards of Africa may occur rather than jumps into quite different climatic environments. However, some nonlinear responses may appear where independent variables, such as interspecific interactions that are indifferent to changing climate parameters may influence the species. This research output suggests that *Anopheles *population abundance, in particular *A. gambiae*, towards the eastern and southern parts of Africa may increase considerably with the projected climate scenario 1 and 3 than with scenario 2. This means that Africa's temperature rise by 0.1°C per degree latitude will provoke less changes on the malaria vectors distribution than an Africa wide temperature increase of 2°C to 4°C.

In the past years, a member of the *A. gambiae *complex demonstrated its ability of taking advantage of modern means of transport to become established in regions (north-eastern Brazil) outside its normal area of geographical distribution as was illustrated during the model validation. The present research has guided us in suggesting that *A. gambiae *is most likely to be the species responsible for the 1930's malaria outbreak in Brazil. Another range extension of a member of the *A. gambiae *complex occurred in about 1942 into Upper Egypt [21]. As in Brazil, its arrival was heralded by severe epidemics of malaria. As already mentioned above, in the course of that event, scientists were still unaware that *A. gambiae *was a complex. In the light of this study, the probable species concerned with the range expansion in Upper Egypt could have been the *A. arabiensis*. This argument is based on the fact that the northern limits of distribution for *A. arabiensis *in the Nile Valley are normally in the northern part of the Sudan. Seasonal climate fluctuation may easily favour the species invasion in neighbouring regions, and it is also possible that similar unrecorded invasions have occurred in the past and may continue to occur in the future.

No claim is being made that climate alone is the main determinant factor for *Anopheles *species existence. However, it is the only factor with available global data that provides an initial estimate of the potential range of *Anopheles *species. The present study should rather be seen as an illustration of the substantial influence, which the direct effects of climate change may constitute to malaria vectors redistribution in Africa. The results presented here should be interpreted as an indication of the sensitivity of malaria vectors to global climatic changes, particularly temperature rise. More elaborate future risk assessments of climate change on malaria vectors in Africa will ultimately need to include: 1) the global climate-based analysis, 2) local socioeconomic and environmental factors, 3) biotic processes such as competition and predation. These new components will add to comprehension and provide better guidance in taking sustainable and efficient preventive measures for malaria control. Other climate-related disasters such as floods are not to be neglected. This type of event may change the dynamics of malaria vectors by creating conditions that allow proliferation and enhance mosquito-human contact.

It should be emphasized that, the presence of malaria vectors within a region does not automatically translate to the disease. There are many countries in the world where other *Anopheles *species capable of transmitting malaria lived, however the disease has disappeared. In some countries, such as the USA, this was attributed to population shift from rural to urban areas, improved housing and nutrition, better water management and living standards and greater access to medical service [22]. If by a magic coup, all these conditions are met in the African continent, malaria could as well be reduced. It is important to point out that human activities and behaviour is crucial for malaria transmission. For example, the daily life patterns, the location of homes in relation to mosquitoes breeding sites, the type of materials used to build and the structure of houses, are key determinant factors for transmission of this deadly disease.

## Conclusion

It is essential to recognize here that there are major malaria control efforts underway at present in Africa; and indicators demonstrate remarkable success in malaria control in selected countries in West, East and Southern Africa [23]. However, history has shown that, there is a challenge in maintaining control and, therefore, understanding the role of climate and environment on the potential for future disease risk is highly valuable. These results have increased the understanding of the future geographical distribution of the *Anopheles *species most responsible for malaria transmission in Africa. They will permit efficient planning of strategies for targeted control or further sampling, detection of competitive interactions, identification of areas in which particular species are potentially involved in malaria transmission, and estimation of risk of introduction to other parts of the world. Informed decisions and actions on these matters is imperative with the changing climate where even traditionally unsuitable areas for *Anopheles *mosquitoes and malaria have been invaded by the vector and the disease.

## Competing interests

The authors declare that they have no competing interests.

## Authors' contributions

HEZT developed the research proposal, participated in its design and drafted the manuscript. RYMK participated in the implementation and validation and helped to draft the manuscript. PZY provided the funding, coordinated the work and participated in the manuscript write up. All authors read and approved the final manuscript.
